# Supplementary material for: Electrically reversible cracks in an intermetallic film controlled by an electric field
Source: Nat Commun. 2018 Jan 3;9:41. doi: 10.1038/s41467-017-02454-8 (PMC5752679; doi:10.1038/s41467-017-02454-8)
Supplement: Supplementary file 1 — Supplementary Information [file 41467_2017_2454_MOESM1_ESM.pdf]

Supplementary Note 1

First-principles calculations of MnPt by VASP code. Starting from a 4-atom $L1_0$ structure of MnPt (*i.e.*, Mn_2Pt_2), we assume that the Mn site can be occupied by either the spin up or the spin down Mn atoms (Mn^+ or Mn^-). We tested all the possible 4-, 8-, and 12-atom supercells. Figure S1 shows the relative energy as a function of equilibrium volume of MnPt for these tests.

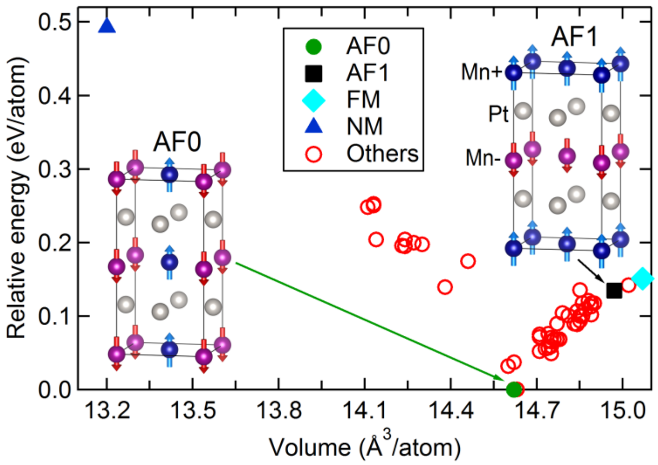

Supplementary Figure 1| Relative energy as a function of equilibrium volume of MnPt. AF, FM and NM stand for antiferromagnetic, ferromagnetic and nonmagnetic structures, respectively.

The present first-principles calculations indicate that:

1. The AF0 is the lowest energy structure (Fig. S1), while the NM is the highest energy structure.
2. Among all the AF structures, the lower energy structure should be the one with the maximum entropy distribution[1] within the Mn sublattice, *i.e.*, the nearest Mn atoms of Mn+ is Mn-, and the nearest Mn atoms of Mn- is Mn- (see AF0 and AF1).
3. If all the Mn atoms in MnPt structure possess larger magnetic moments (> 3.0 μ_B), then these structures have higher equilibrium volumes with respect to that of AF0. We call these structures the group 1 structures. For the group 1 structures, the FM has the highest (or higher) energy.
4. If one or more Mn atoms in MnPt have smaller magnetic moments (< 1.0 μ_B), then these structures possess lower equilibrium volumes (with respect to AF0), we call these structure as the group 2 structures. Specially, the NM is a structure with the magnetic moment of zero for all Mn atoms.
5. In principle, group 1 structures have lower energies than those of the group 2 structures, indicating that the MnPt phase shows an anti-Invar behavior at high temperatures due to magnetism.
6. The defects of Mn atoms in Mn site (Mn sublattice) will cause a great increase of energy, *i.e.*, the anti-site of spin up Mn (Mn+) and spin down Mn (Mn-). For example, the energy of AF1 (see Fig. S1, due to anti-site of Mn+ and Mn- within the Mn sublattice) is comparable with that of the FM structure. Hence, the defects within Mn sublattice due to

site change of Mn+ and Mn- will cause the more stability of the FM with respect to the AF structure.

7. We also tested other defects, such as Mn vacancy, Pt vacancy, and anti-site of Mn and Pt. These defects will decrease the energy difference between the AF0 phase and the FM phase, but, a significant decrease of their energy difference will be the Mn defects (such as anti-site of Mn+ and Mn-) within the Mn sublattice as shown in Fig. S1.

The calculated structural, elastic and magnetic parameters for the three different magnetic structures are shown in Tabs. S1-S3.

Supplementary Table 1| Calculated mechanical and magnetic parameters. Equilibrium properties of three structures from the 4-parameter Birch-Murnaghan equation of state (EOS) fitting[2] after first-principles calculations.

Structure	Volume ($Å^3$/atom)	Relative energy (eV/atom)	Bulk modulus (GPa)	B' (the first derivate of bulk modulus with respect to pressure)	Magnetic moment of Mn (μ_B/atom)
AF0	14.68	0.000	174.8	5.08	3.7
AF1	15.06	0.132	157.4	4.53	3.8
FM	15.17	0.147	164.0	3.97	3.8

Supplementary Table 2| Calculated elastic constants. Single-crystal elastic properties (C_{ij}) of AF0 and FM MnPt (unit is GPa) calculated by the strain-stress method according to first-principles calculations[3].

Structure	C_{11}	C_{12}	C_{13}	C_{33}	C_{44}	C_{66}
AF0	264	115	140	283	123	117
FM	244	108	149	232	99	87
FM[a]	257	89	149	224	100	71

[a] Prediction according to Materials Project database[4].

Supplementary Table 3| Calculated bulk modulus, shear modulus, and Young's modulus. Aggregative elastic properties of MnPt from the C_{ij} values in Tab. S2, where the Hill approach[5] was employed. These properties include bulk modulus (B), shear modulus (G), Young's modulus, and Poisson ratio (v). Note bulk moduli from equation of state (EOS) fitting are 174.8 and 164.0 GPa for AF0 and FM, respectively, which is slightly smaller than the ones from the C_{ij} values due to more relaxations in EOS fitting[2].

Structure	B_H (GPa)	G_H (GPa)	E_H (GPa)	B/G	v
AF0	177.3	96.5	245.1	1.84	0.27
FM	169.8	72.8	191.2	2.33	0.31
FM[a]	167.6	71.5	187.8	2.34	0.31

[a] Prediction from the Materials Project database[4].

Supplementary Note 2

Mechanical tests of MnPt films. In order to assess the mechanical behavior of MnPt films, nanoindentation was utilized. A three-sided pyramidal Berkovich tip with a radius of about 100 nm was used to indent the surface of a 35 nm thick MnPt film on a silicon substrate on a Hysitron TriboScope (Minneapolis, MN, USA). Thirty indents were performed within a maximum displacement range between 5-80 nm (Fig. S2a). The representative load-displacement curves illustrate permanent plastic deformation and no pop-ins that would indicate thin film fracture events. From the initial unloading slopes of the load-displacement curves the elastic modulus using the Oliver-Pharr method[6] was calculated as well as the hardness (Fig. S2b). The mechanical properties of the MnPt film should be taken near the surface (5-30 nm) to avoid influence from the substrate. The elastic modulus of the MnPt film was measured to be approximately 155-160 GPa (close to the calculated bulk modulus B = 177 GPa) and the hardness was 5 GPa. A significant increase in the hardness after 30 nm of displacement is an indication of the substrate influence on the measured value.

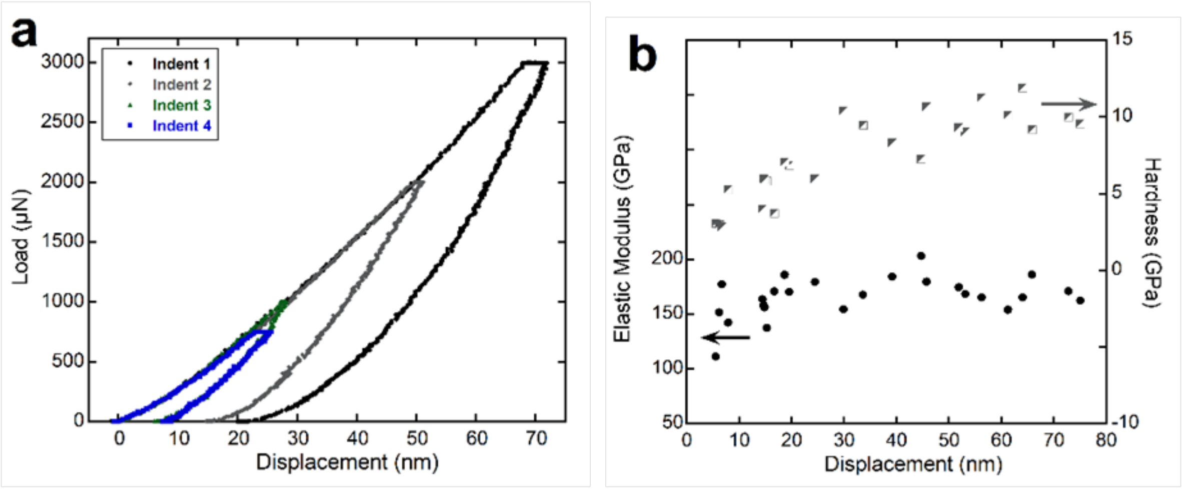

Supplementary Figure 2| Nanoindentation results. a, Representative load-displacement curves made using a Berkovich tip illustrating permanent plastic deformation into the MnPt film on silicon. **b,** Calculated elastic modulus and hardness of the MnPt film in the indented displacement range.

Supplementary Note 3

Pugh's criterion (B/G ratio) to judge brittleness/ductility. The Pugh's criterion based on the B/G ratio is a simple model to judge the brittleness or ductility of a solid material, since the bulk modulus B can be considered as the resistance to fracture and the shear modulus G the resistance to plastic deformation. A critical B/G ratio (around 1.75) to separate the brittle and ductile materials was implied by Pugh[7] based on *fcc* metals with high melting point. The microscopic origin of this empirical parameter (B/G = 1.75) can be understood from an isotropic cubic crystal with Cauchy relations $C_{11} = 3C_{12}$ and $C_{12} = C_{44}$, resulting in a B/G value of 1.67[8]. The critical value (B/G = 1.75) has been widely used in first-principles community for various materials, see such as the work done by Ravindran *et al.*[8] in 1998 for the orthorhombic TiS_2 by considering the ductile Ti (B/G = 2.47) and the brittle Si (B/G = 1.49).

Although the criterial B/G ratio (1.75) was driven from *fcc* metals with high melting point, we found it works for $L1_0$-type compounds by considering the brittle TiAl[9] with B/G = 1.64 and the ductile CoPt[10] with B/G = 2.00 (data from the Materials Project database[4], see Tab. S4). It is worth mentioning that the criterial B/G = 1.75 don't work for all materials as pointed by, for example, Pugh[7], Gandhi and Ashby[11].

The present MnPt with B/G = 1.84 should be near-brittle within the category of $L1_0$-type compounds, and MnPt is more brittle than its constituent elements, *i.e.*, the metastable *fcc* Mn with B/G = 2.14 and *fcc* Pt with B/G = 4.98, see Tab. S4. It is further noticed that the brittleness of MnPt stems from the antiferromagnetic Mn atoms by considering the B/G ratios for the FM and AF MnPt (2.32 *vs*. 1.84, see Tab. S4).

Supplementary Table 4| Calculated bulk modulus *B* and shear modulus *G* as well as the *B/G* ratio in the Hill approach. These elastic properties were taken from the Materials Project database[4] and the present work. All the available $L1_0$-type compounds (space group *P4/mmm*) with elastic properties together with the *fcc* Mn and *fcc* Pt are listed.

Formula	Materials Project ID	Space group	*B* (GPa)	*G* (GPa)	*B/G*	Note
MnAl	mp-771	*P4/mmm*	133	84	1.58	
TiGa	mp-2767	*P4/mmm*	115	72	1.60	
TiAl	mp-1953	*P4/mmm*	115	70	1.64	Brittle [6]
CoPt	mp-949	*P4/mmm*	216	108	2.00	Ductile [7]
FeNi	mp-2213	*P4/mmm*	187	92	2.03	
FePt	mp-2260	*P4/mmm*	201	92	2.18	
ZnNi	mp-429	*P4/mmm*	146	63	2.32	
MnPt[a]	mp-1670	*P4/mmm*	168	72	2.33	
MnPt[a]		*P4/mmm*	170	73	2.32	This work
MnPt[b]		*P4/mmm*	177	96	1.84	This work
NiPt	mp-945	*P4/mmm*	213	91	2.34	
NbIr	mp-1359	*P4/mmm*	259	105	2.47	
ZnPd	mp-1652	*P4/mmm*	139	55	2.53	
CdPd	mp-1696	*P4/mmm*	110	42	2.62	
MnPd	mp-238	*P4/mmm*	134	51	2.63	
MgIn	mp-2313	*P4/mmm*	40	15	2.67	
ZnPt	mp-894	*P4/mmm*	172	63	2.73	
NbRh	mp-1963	*P4/mmm*	220	79	2.78	
TiIr	mp-1235	*P4/mmm*	228	81	2.81	
CdPt	mp-1194	*P4/mmm*	137	48	2.85	
HgPd	mp-2685	*P4/mmm*	115	35	3.29	
TaRu	mp-1601	*P4/mmm*	252	73	3.45	
TiRh	mp-2583	*P4/mmm*	190	49	3.88	
ZrHg	mp-2510	*P4/mmm*	102	24	4.25	
NbRu	mp-432	*P4/mmm*	237	53	4.47	

CuAu	mp-522	*P4/mmm*	135	28	4.82	
fcc Mn	mp-8634	$Fm\bar{3}m$	280	131	2.14	
fcc Pt	mp-126	$Fm\bar{3}m$	244	49	4.98	

Supplementary Methods

First-principles calculations. In the present work, all density functional theory (DFT) based first-principles calculations were performed by VASP code[12] with the electron-ion interaction described by the projector augmented wave method[13] and the exchange-correlation functional described by the generalized gradient approximation[14]. The $L1_0$-type low energy antiferromagnetic (AFM) and the ferromagnetic (FM) structures were employed for MnPt, see details reported previously[15]. Single crystal elastic constants of AFM and FM MnPt were calculated by an efficient strain-stress method[3] with the employed non-zero strains of ±0.01. The same VASP settings as used previously[15], 5,000 (or 8,000) *k*-points per reciprocal atom and plane wave energy cutoff of 270 eV (or 337 eV) were used for structural relaxations (or final static calculations) to calculate elastic constants using VASP. Note that the final static calculations were performed by the tetrahedron method with Blöchl correction[16] for accurate stress results.

In addition, the mentioned energy vs. volume equation of state (EOS) fittings were performed by a 4-paramter Birch-Murnaghan equation[2] in terms of about eight energy vs. volume first-principles data points for each structure. Based on the obtained single crystal elastic constants, aggregative elastic properties were estimated using the Hill approach[5], including bulk modulus (*B*), shear modulus (*G*), Young's modulus, and Poisson ratio (*v*).

Supplementary References:

1. Shang, S., Wang, Y., Wang, W. Y., Fang, H. & Liu, Z.-K. Low energy structures of lithium-ion battery materials $Li(Mn_xNi_xCo_{1-2x})O_2$ revealed by first-principles calculations. *Appl. Phys. Lett.* **103,** 53903 (2013).

2. Shang, S. L., Wang, Y., Kim, D. & Liu, Z. K. First-principles thermodynamics from phonon and Debye model: Application to Ni and Ni_3Al. *Comput. Mater. Sci.* **47,** 1040–1048 (2010).

3. Shang, S. L., Wang, Y. & Liu, Z. K. First-principles elastic constants of α- and θ-Al_2O_3. *Appl. Phys. Lett.* **90,** 101909 (2007).

4. Jain, A. *et al.* Commentary: The Materials Project: A materials genome approach to accelerating materials innovation. *APL Mater.* **1,** 11002 (2013).

5. Hill, R. The elastic behaviour of a crystalline aggregate. *Proc. Phys. Soc. Sect. A* **65,** 349–354 (1952).

6. Oliver, W. C. & Pharr, G. M. An improved technique for determining hardness and elastic modulus using load and displacement sensing indentation experiments. *J. Mater. Res.* **7,** 1564–1583 (1992).

7. Pugh, S. F. XCII. Relations between the elastic moduli and the plastic properties of polycrystalline pure metals. *Philos. Mag.* **45,** 823–843 (1954).

8. Ravindran, P. *et al.* Density functional theory for calculation of elastic properties of orthorhombic crystals: Application to $TiSi_2$. *J. Appl. Phys.* **84,** 4891–4904 (1998).

9. Booth, A. S. & Roberts, S. G. The brittle-ductile transition in γ-TiAl single crystals. *Acta Mater.* **45,** 1045–1053 (1997).

10. Greenbery, B. A. *et al.* Optimised mechanical properties of ordered noble metal alloys. *Platin. Met. Rev.* **47,** 46–58 (2003).

11. Gandhi, C. & Ashby, M. F. Fracture mechanism maps for materials which cleave: F.C.C., B.C.C. and H.C.P. metals and ceramics. *Acta Metall.* **27,** 1565–1602 (1979).

12. Kresse, G. & Furthmüller, J. Efficiency of ab-initio total energy calculations for metals and semiconductors using a plane-wave basis set. *Comput. Mater. Sci.* **6,** 15–50 (1996).

13. Kresse, G. & Joubert, D. From ultrasoft pseudopotentials to the projector augmented-wave method. *Phys. Rev. B* **59,** 1758–1775 (1999).

14. Perdew, J. P., Burke, K. & Ernzerhof, M. Generalized gradient approximation made simple. *Phys. Rev. Lett.* **77,** 3865–3868 (1996).

15. Liu, Z. *et al.* Epitaxial growth of intermetallic MnPt Films on oxides and large exchange bias. *Adv. Mater.* **28,** 118–123 (2016).

16. Blöchl, P. E., Jepsen, O. & Andersen, O. K. Improved tetrahedron method for Brillouin-zone integrations. *Phys. Rev. B* **49,** 16223–16233 (1994).
